# Telemonitoring in adolescents with inflammatory bowel disease: a systematic review

**DOI:** 10.1007/s00431-025-06341-z

**Published:** 2025-08-05

**Authors:** Mike P. T. Kusters, Marleen Bouhuys, Robin W. M. Vernooij, Linde F. Huis in ’t Veld, Johan E. van Limbergen, Bada Yang, Patrick F. van Rheenen

**Affiliations:** 1 Cochrane Netherlands, Julius Center for Health Sciences and Primary Care, University Medical Centre Utrecht, Utrecht University, Utrecht, the Netherlands; 2 Department of Pediatric Gastroenterology, Hepatology and Nutrition, University of Groningen, University Medical Centre Groningen, Beatrix Children’s Hospital, PO Box 30001, 9700 RB Groningen, the Netherlands; 3 Department of Pediatrics, Frisius Medical Centre, Leeuwarden, the Netherlands; 4 Department of Nephrology and Hypertension, University Medical Center Utrecht, Utrecht, the Netherlands; 5 Department of Pediatric Gastroenterology and Nutrition, Emma Children’s Hospital, Amsterdam University Medical Centers, Amsterdam, the Netherlands

**Keywords:** Crohn disease, Colitis, ulcerative, Pediatrics, Adolescence

## Abstract

Telemonitoring could be an alternative for standard face-to-face consultations. In adults with inflammatory bowel disease (IBD), telemonitoring has been associated with improved quality of life (QoL) and reduced costs. We aimed to assess whether telemonitoring of adolescents with IBD is non-inferior to standard care in controlling disease activity. We searched Medline and Embase up to June 15, 2025, for randomized trials comparing the effect of telemonitoring and standard care on disease activity in adolescents with IBD. Secondary outcomes were QoL, costs, patient satisfaction, patient adherence, face-to-face contacts, and emergency room attendances or hospitalizations. Risk of bias and certainty of evidence were assessed using the Risk of Bias 2 tool and GRADE, respectively. Data was synthesized and reported in accordance with the Synthesis Without Meta-analysis (SWiM) reporting guideline and the Cochrane Handbook. Three studies (309 patients) were identified. Risk of bias was judged “high” for one study and as “some concerns” for the remaining two studies. We found that telemonitoring may not worsen disease activity (low-certainty evidence) and may result in little to no improvement in QoL (low-certainty evidence) compared to standard care. Telemonitoring likely results in a slight cost reduction (moderate- certainty evidence). *Conclusion*: Telemonitoring may be non-inferior to standard care in controlling disease activity for adolescents with IBD.

**Table:** 

**What is Known:** • *Studies comparing telemonitoring with standard follow-up in adults with inflammatory bowel disease (IBD) did not find a difference in disease activity or the occurrence of flares between these strategies.* • *Telemonitoring could improve quality of life and reduce costs and health care utilization in adults.*
**What is New:** • *This systematic review exclusively reviews the effect of telemonitoring on adolescents with IBD.* • *Based on limited evidence, following adolescents with IBD with telemonitoring appears to be safe and may reduce the number of outpatient visits and societal costs.*

**Supplementary Information:**

The online version contains supplementary material available at 10.1007/s00431-025-06341-z.

## Introduction

Inflammatory bowel disease (IBD), including Crohn’s disease and ulcerative colitis, is a chronic immune-mediated disease that is characterized by periods of remission and periods of disease flares. Because of the fluctuating disease course, regular monitoring of disease activity is required, followed by therapy adjustment in case of an imminent flare. Patient follow-up usually consists of prescheduled face-to-face consultations at the outpatient IBD clinic. In numerous consultations, the disease may remain inactive and treatment will be unaltered [1].

Telemonitoring in IBD encompasses remote measurement of disease markers, such as symptom scores or inflammatory markers in feces or blood. In case of abnormal findings, treatment can be adjusted promptly. In recent years, many clinics developed a telemonitoring care pathway in addition to face-to-face consultations to manage their adult patients with IBD. Telemonitoring was strengthened during the COVID-19 pandemic [2].

Several systematic reviews and meta-analyses have been published about the effects of telemonitoring care as compared to standard care in adults with IBD [3–8]. In the adult IBD population followed with telemonitoring, disease control was well maintained and probably not different from standard care [3–8]. However, these findings cannot be extrapolated directly to adolescents with IBD. For example, while the cognitive ability and reasoning capacity of adolescents resembles that of adults, they may experience internal conflicts related to autonomy, leading to reduced adherence to the monitoring protocol and increased likelihood of adverse outcomes [9]. We carried out a systematic review to evaluate whether using telemonitoring is non-inferior to standard care in controlling disease activity in adolescents with IBD.

## Materials and methods

### Search strategy and selection criteria

For this systematic review, we searched MEDLINE (via OVID) and Embase (via Embase.com) for randomized controlled trials (RCTs) from database inception to June 15, 2025, without language restrictions. The detailed search strategies are provided in the supplementary material (Table S1 and S2). The protocol of this review was registered on PROSPERO (CRD42022348872). Since this is a systematic review of published studies, ethical approval by an ethics committee was not applicable.

We included RCTs including children and adolescents up to 18 years of age with IBD, receiving any kind of telemonitoring (e.g., web-, mobile-, and phone-based). Studies were included if telemonitoring was compared to standard care and if one or more of the following outcomes were reported: disease activity, quality of life (QoL), costs, patient satisfaction, patient adherence, face-to-face clinician-patient outpatient contacts, and a composite outcome consisting of unplanned emergency room attendance, surgery, or hospitalizations. We did not define these outcome measures a priori but used definitions reported in included studies.

We first screened titles and abstracts and then reviewed full text reports in duplicate (i.e., by two reviewers independently). Disagreements were resolved by a third reviewer. Data extraction and risk-of-bias assessments were performed by one reviewer and checked by two other reviewers. Here, disagreements were resolved through consensus.

### Data extraction and assessment of study quality

Data was extracted from study reports and available supplementary appendices. Data was extracted for study design, population characteristics (e.g., number of participants, age, sex, disease type, and proportion with active disease), characteristics of intervention and comparator (e.g., telemonitoring type, intervention interval, and follow-up), outcomes, source of funding, and conflict of interests. Risk of bias was assessed using the revised Cochrane risk-of-bias tool for randomized trials (RoB 2) [10]. Certainty of the body of evidence was assessed using GRADE (Grading of Recommendations Assessment, Development, and Evaluation) [11]. The PRISMA 2020 checklist is available in Table S8.

### Data analysis

After finalizing the protocol and gathering all outcome data, we decided to split the outcome domain “patient-reported experience and engagement” into “patient satisfaction” and “patient adherence.” Heterogeneity in reported outcomes precluded meta-analyses. Instead, we described the results and assessed heterogeneity according to the Synthesis Without Meta-analysis (SWiM) reporting guideline [12], and the different scenarios when meta-analysis is not possible provided by the Cochrane Handbook for Systematic Reviews of Interventions [13].

Where possible, studies were primarily grouped and ranked based on their risk-of-bias ratings and secondarily on the number of randomized participants. A priori, we established no hierarchy in telemonitoring type or outcome measurements or tools within each overarching outcome domain. Effect estimates such as mean difference (MD) and risk ratio (RR) were calculated using Review Manager 5.4 if data allowed [14]. Medians with interquartile ranges (IQRs) and/or minimum and maximum values were transformed to means and standard deviations (SD) using formulas by Wan et al. [15] if required for the chosen synthesis method. Heterogeneity across studies was assessed using informal methods (e.g., structured description of tables and plots which allowed for visual assessment of heterogeneity of reported effect directions across studies). We did not define thresholds for non-inferiority of telemonitoring versus standard care in advance.

### Role of funding source

This study was funded by the acceleration program (“Versnellingsprogramma Duidingen”) by ZonMw and the Health Care Evaluation and Appropriate Use program (ZE&GG). The funder of the study had no role in study design, data collection, data analysis, data interpretation, or writing of the report.

## Results

### Study selection

**Fig. 1:**
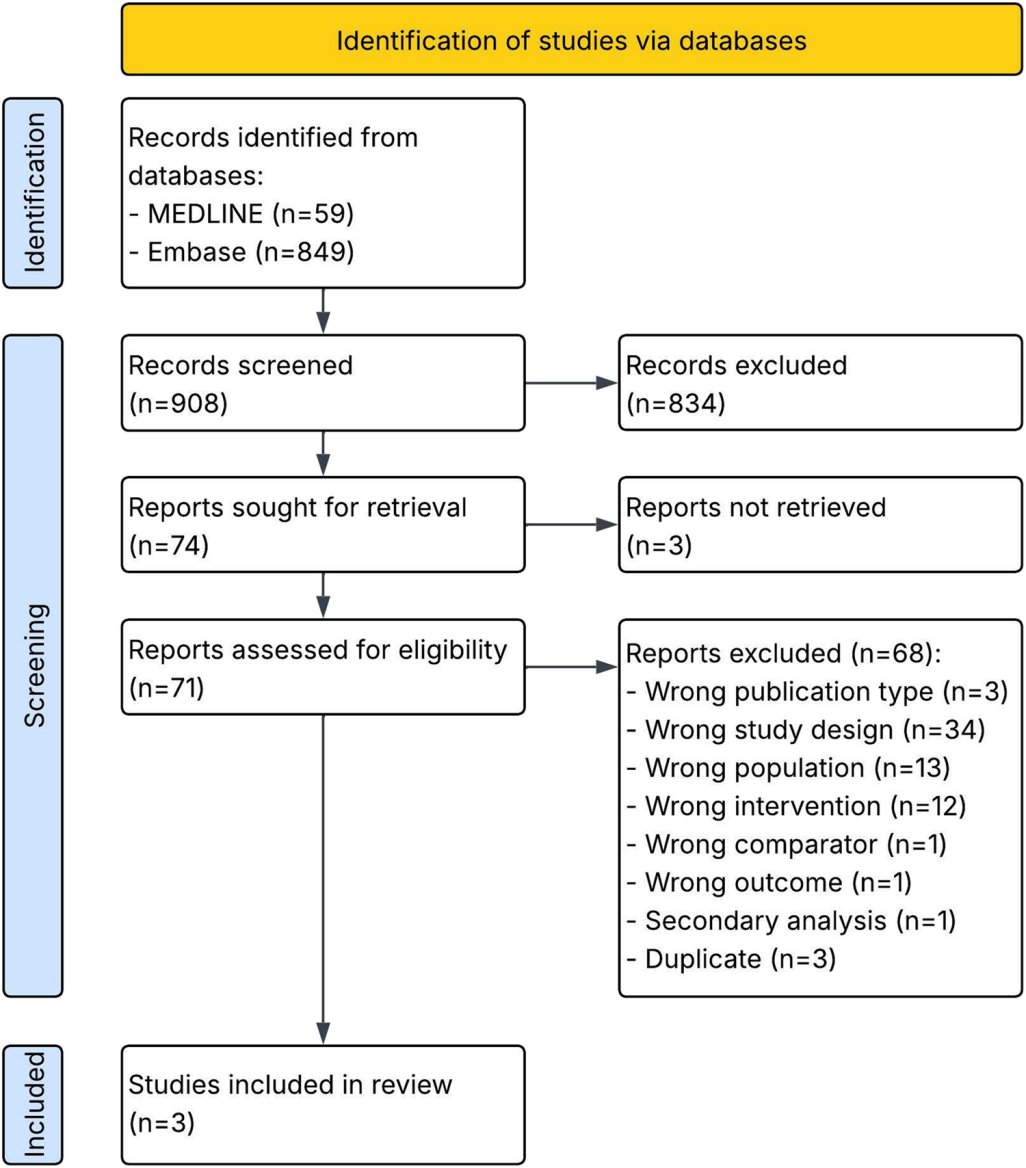
PRISMA 2020 flow diagram [19]

The search strategy identified 908 unique records, of which 74 were assessed based on full text, and 3 records (3 unique studies) were included (Fig. 1) [16–18]. The most frequent reasons for exclusion based on full-text assessment were wrong study design and wrong population (Table S3).

### Description of studies

Tables 1 and S4 contain an overview of characteristics of included studies. All included studies were designed as parallel RCTs, comparing telemonitoring versus standard care in adolescents with IBD. The total number of randomized participants ranged between 53 and 170. The proportion of females across the studies ranged between 37 and 58%. Study populations included adolescents with an age range between 11.2 and 17 years. In the studies by Akobeng et al. and Heida et al., all participants were in clinical disease remission at baseline [16, 17]. In the study by Carlsen et al., 57% had active disease at baseline [18].

The telemonitoring intervention was phone-based [17] or web-based [16, 18]. Monitoring intervals were monthly [18], tailored to risk stratum [16], or not reported [17]. Monitoring in the control group mainly consisted of planned outpatient visits. The follow-up period was 12 [18] or 24 [16, 17] months.

All studies provided measurements of the primary outcome, disease activity (disease flares, treatment intensification), and all studies measured QoL with the IMPACT(-III) questionnaire [20–22]. Data was reported for all other outcomes, but not in all included studies. The characteristics of each study and the direction of effect per outcome are summarized in Table 1. More information on the reported study outcomes and results is available in Tables S5 and S6.

**Table 1:** Study characteristics and adapted effect direction plot based on Boon and Thomson [23]

Study characteristics								Direction of effect						
Study	Risk of bias (RoB2)	Randomized participants	Telemonitoring-based type	Disease type (%)	Active disease at enrolment (%)	Mean (SD) or median [IQR] age in years at enrolment	Follow-up (months)	Disease activity	QoL	Costs	Patient satisfaction	Patient adherence	Face-to-face outpatient contacts	Unplanned ER/surgery/hospitalizations
Heida et al. [16]	Some concerns	170	Web	UC: 52	0	I: 15[12-16]	12	◇	△	△	✸	▼	▲	◇
				CD: 48		C: 15[13-17]								
Akobeng et al. [17]	Some concerns	86	Phone	UC: 17	0	I: 13·9[12·1-15·9]	24	△	△	▲	◇	◇		
				CD: 83		C:13·8[11·2-15·3]								
Carlsen et al. [18]	High	53	Web	UC: 60	57	I: 15·1 (1·82)	24	◇	◇			◇	◇	△
				CD: 40		C: 14·7 (2·11)								

△ non-significant result in favor of the telemonitoring group; ▲ significant result in favor of the telemonitoring group; ▼significant result in favor of standard care; ◇ no difference/mixed effects/conflicting findings; ✸ positive non-comparative result in the telemonitoring group; *C* control group, *CD* Crohn’s disease, *ER* emergency room, *I* intervention group, *QoL* quality of life, *RoB2* Cochrane revised tool to assess the risk of bias in randomized trials, *SD* standard deviation, *UC* ulcerative colitis

Regarding risk of bias, there were some concerns for two studies [16, 17] due to risk of bias in the measurement of the outcome. One study was at high risk of bias due to shortcomings in the measurement of the outcome and due to missing outcome data (Table S7) [18]. Assessments of the certainty of evidence are summarized in Table 2.

**Table 2:** Certainty of evidence

Outcome measure	Certainty of evidence (GRADE)
Disease activity	(low)
Quality of life	(low)
Costs	(moderate)
Patient satisfaction	(low)
Patient adherence	(low)
Face-to-face outpatient visits	(low)
Emergency room visits, surgery, and hospitalizations	(moderate)

*GRADE* Grading of Recommendations Assessment, Development, and Evaluation

### Disease activity

All three included studies reported a measure of disease activity [16–18].

Akobeng et al. found a non-significantly lower number of relapses, defined by Pediatric Ulcerative Colitis Activity Index (PUCAI) or abbreviated Pediatric Crohn’s Disease Activity Index (aPCDAI) score(s) > 15, in the telemonitoring group compared to standard care (1 per 44 patients (2%) versus 4 per 42 patients (10%), 1 study, some concerns of bias). RR for disease relapse was 0.24 (95% confidence interval [CI] 0.03 to 2.05, *p* = 0.20) [17].

Heida et al. found that the cumulative incidence of disease flares, defined as disease activity necessitating therapy intensification, was similar between telemonitoring and control (28 per 84 patients (33%) versus 29 per 86 patients (34%), 1 study, some concerns of bias). RR for disease flare was 0.99 (95% CI 0.65 to 1.51, *p* = 0.96). The study did not find a difference in time-to-flare between groups (*p* = 0.932) [16]. Carlsen et al. reported that the time to first step-up in treatment intensity (escalating dose or change/addition of a more potent drug) did not differ between groups (*p* = 0.53, 1 study, high risk of bias) [18].

Certainty of evidence for disease activity was low due to risk of bias (lack of blinding of outcome assessors and missing outcome data) and imprecision (low number of events and participants and wide confidence intervals around the reported effects that do not preclude inferiority of telemonitoring).

### Quality of life

Pediatric IBD health-related quality of life (QoL) was assessed by all three included studies [16–18] with the IMPACT-III QoL questionnaire [20–22]. Across the included studies, multiple score ranges were used (0 to 100 [16]; 0 to 140 [16]; 35 to 175 [18]).

All studies reported a non-inferior effect of telemonitoring versus standard care on QoL. Two out of three studies reported results in favor of telemonitoring [16, 17]. The effect estimate in Akobeng et al. was a MD of 8.7 (95% CI 1.1 to 16.3, *p* = 0.03, 1 study, some concerns of bias) in favor of telemonitoring. The MD was 5.7 (95% CI − 2.9 to 14.3, *p* = 0.19) when adjusted for baseline outcome and disease type [17].

Heida et al. reported a non-significant improvement in mean change QoL score from baseline in the telemonitoring group versus the control group (1.3 versus − 0.3 respectively, *p* = 0.27, 1 study, some concerns of bias) [16].

Carlsen et al. merely reported that there was no difference in QoL between groups (1 study, high risk of bias) [18].

Certainty of evidence for QoL was low, due to risk of bias (lack of blinding of outcome assessors and missing outcome data) and imprecision (low number of participants and wide confidence intervals around the reported effect that do not preclude inferiority).

### Costs

Two out of three studies reported measures of cost [16, 17].

Akobeng et al. found lower mean consultation costs in the telemonitoring group than in the control group, 35.4 versus 51.1 British pounds respectively (MD − 15.7 pounds; 95% CI − 11.8 to − 19.6; *p* < 0.001; 86 participants (44 telemonitoring, 42 control group); 1 study; low risk of bias) [17].

Heida et al. reported in a trial-based cost-effectiveness analysis that the annual cost savings of telemonitoring, compared to standard care, was 89 euros per person. In participants compliant to the protocol (response to ≥ 80% of automated alerts in the telemonitoring group or sending in ≥ 2 stool samples for calprotectin measurement in the control group), these savings were 360 euros per person (170 included participants, of whom 120 compliant (48 telemonitoring group (57%), 72 control group (84%)); 1 study; low risk of bias). This cost-effectiveness analysis incorporated all direct and indirect medical and non-medical costs [16].

Certainty of evidence for costs was moderate due to imprecision (low number of participants and studies).

### Patient satisfaction and adherence measures

Satisfaction and/or adherence measures were reported in all three included studies [16–18].

Two studies described measures of patient satisfaction [16, 17]. Heida et al. reported that 71% of 59 intervention group participants wished to continue with home telemonitoring care and that 96% considered home telemonitoring as timesaving [16]. Akobeng et al. reported no difference between groups regarding patient satisfaction with consultations, using the child-modified Consultation Satisfaction Questionnaire (CSQ-child) (MD 1.00; 95% CI − 1.25 to 3.25; *p* = 0.38). After adjusting for baseline outcome and disease type, the MD was 0.59 (95% CI − 2.05 to 3.2; *p* = 0.65) [17].

Certainty of evidence for patient satisfaction was low, due to risk of bias (lack of blinding of outcome assessors) and imprecision (low number of participants and studies).

All three studies reported on patient adherence measures [16, 18]. Carlsen et al. described no difference between groups regarding medication adherence (Medication Adherence Report Scale [MARS]), adjusting for age and time from diagnosis (53 participants, 27 telemonitoring, and 26 control group participants; 1 study; high risk of bias) [18]. Akobeng et al. reported results on consultation adherence. The proportion of attended consultations was 67% with telemonitoring (telephone consultations) and 71% in the control group (face-to-face consultations) (RR for attendance 1.06 (0.78 to 1.43); *p* = 0.71; 86 participants (44 telemonitoring, 42 control group); 1 study; low risk of bias) [17]. Heida et al. reported results on protocol compliance: 57% of 84 participants in the telemonitoring group was compliant to the study protocol (defined as responding to ≥ 80% of automated alerts), whereas in the standard care group, 84% of 86 participants was compliant (sending in ≥ 2 stool samples for calprotectin measurement) (RR of non-compliance 2.63; 95% CI 1.54 to 4.51; *p* = 0.0004; 170 participants; 1 study; some concerns of bias) [16].

Certainty of evidence for patient adherence was low, due to risk of bias (lack of blinding of outcome assessors) and inconsistency (variability in direction of effect).

### Face-to-face outpatient contacts

All three included studies reported on face-to-face outpatient contacts. Two studies reported a non-inferior effect of telemonitoring versus standard care on this outcome domain, with results in favor of telemonitoring [16, 18].

Carlsen et al. reported fewer planned (38 per 15 patients versus 146 per 18 patients, *p* < 0.001) but slightly more on-demand outpatient visits (47 versus 39, *p* = 0.68) with telemonitoring compared to control. Overall, the telemonitoring group had less outpatient visits compared to the control group (85 versus 185, *p* < 0.0001, 1 study, high risk of bias) [18].

Heida et al. found that telemonitoring resulted in fewer face-to-face encounters with a healthcare provider compared to standard care (300 per 84 patients versus 328 per 86 patients (mean 3.6 versus 4.3 per patient, *p* < 0.001), 1 study, low risk of bias) [16].

Face-to-face outpatient contacts were only assessed for the control group in Akobeng et al. with a median per person of 3 [IQR 2 to 4] (30 per 42 patients, 1 study, low risk of bias) [17].

Certainty of evidence for face-to-face outpatient contacts was low, due to risk of bias (missing outcome data) and imprecision (low number of events and wide confidence intervals around the reported effect).

### Unplanned ER visits, surgery, and hospitalizations

Two studies reported results for unplanned ER visits and hospitalizations [16–18]. No results on unplanned surgery were reported.

Carlsen et al. found no difference in the median number of acute ER visits or hospitalizations between the telemonitoring and control groups, with a median of, respectively, 0 [IQR 0 to 0] and 0 [IQR 0 to 1] events (*p* = 0.13). However, the study reported overall fewer acute ER visits or hospitalizations in the telemonitoring group (3 visits per 15 patients versus 10 visits per 18 patients in total, 1 study, high risk of bias) [18].

Heida et al. reported a similar number of emergency consultations, e.g., rapid access to specialist care, between telemonitoring and control groups (respectively 4 per 84 patients and 5 per 86 patients, 1 study, low risk of bias) [16].

Certainty of evidence for unplanned ER visits, surgery, and hospitalizations was moderate, rated down for risk of bias (missing outcome data).

## Discussion

In this systematic review, we included three RCTs with a total of 309 pediatric patients with IBD comparing telemonitoring with standard care regarding disease activity and several other outcomes. Although meta-analyses were not possible, we were able to provide a transparent overview of the evidence by following the SWiM-reporting guideline and the Cochrane Handbook [12, 24]. We found that telemonitoring may not worsen disease activity (low certainty of evidence), telemonitoring may result in little to no improvement of quality of life (low certainty of evidence), and telemonitoring may reduce the total number of face-to-face outpatient visits slightly as compared to standard care (low certainty of evidence). Based on evidence from two studies [16, 17], telemonitoring likely results in a slight reduction of healthcare costs (moderate certainty of evidence) and telemonitoring may not worsen patient satisfaction (low certainty of evidence). Measures of protocol, consultation, and medication adherence showed conflicting results (low certainty of evidence) [16, 18]. Based on evidence from two studies [16, 18], telemonitoring may not result in an increase in unplanned ER visits or hospitalizations, relative to standard care (moderate certainty of evidence). No data were reported on unplanned surgeries.

Our review has several methodological strengths. We were able to provide, for each outcome domain, the certainty of the body of evidence using GRADE methodology. Despite heterogeneity in reporting and the absence of a meta-analysis, we provide a structured summary following recommended Cochrane methods for narratively synthesizing the evidence (the SWiM-reporting guideline and Cochrane Handbook) [12, 24]. Such guidance facilitates complete, transparent syntheses and appropriate conclusions.

We also used the revised Cochrane risk-of-bias tool (RoB 2) [10] instead of the widely used original Cochrane risk-of-bias tool [25]. According to its creators, more outcomes are expected to be assessed as low risk of bias with RoB 2 compared to the original tool, especially in unblinded randomized trials as the ones included in our review [10].

Broad outcome domains with imprecise definition of outcome measures were unavoidable due to lack of standardization and absence of a core outcome set until November 2023 [26]. This heterogeneity in outcome reporting precluded a meta-analysis. We did not pursue attempts to contact study authors for individual participant data to perform meta-analyses. We could not perform formal investigations of heterogeneity or assess risk of publication bias due to the small number of included studies. Lastly, we solely relied on database searching to identify studies, though given the relatively small scope of the pediatric IBD field, we believe it is unlikely that relevant studies were overlooked.

During the COVID pandemic, various forms of telemonitoring were extended to a segment of the adolescent IBD population, ranging from teleconsultations to calprotectin home-testing with written feedback from an online patient portal. These telemonitoring methods persisted beyond the end of the pandemic [27].

Our review findings suggest that telemonitoring may be safe and could be considered an addition to standard face-to-face consultations in pediatric practices, taking into consideration factors such as costs and patient preferences. If no active disease is detected through telemonitoring, the frequency of face-to-face consultations could be reduced. However, most outcome domains were classified as low certainty of evidence using GRADE methodology. Hence, additional research with higher quality evidence is required to strengthen these suggestions.

In summary, our findings indicate that telemonitoring enables timely detection of imminent flares in adolescents with IBD. Between-group differences regarding other outcome domains are either small, inconsistent, or absent. Future trials should aim to use a standardized set of outcomes and outcome definitions to increase comparability and consistency across studies.

## Supplementary Information

Below is the link to the electronic supplementary material.

Supplementary Material 1 (DOCX 1.28 MB)

## Data Availability

No datasets were generated or analysed during the current study.

## Abbreviations

aPCDAI: Abbreviated pediatric Crohn’s disease activity index
CI: Confidence interval
CSQ: Consultation Satisfaction Questionnaire
ER: Emergency room
GRADE: Grading of Recommendations Assessment, Development, and Evaluation
IBD: Inflammatory bowel disease
IQR: Interquartile range
MARS: Medication Adherence Report Scale
MD: Mean difference
PCDAI: Pediatric Crohn’s disease activity index
PUCAI: Pediatric ulcerative colitis activity index
QoL: Quality of life
RCT: Randomized controlled trial
RoB: Risk of bias
RR: Risk ratio
SD: Standard deviation
SWiM: Synthesis Without Meta-analysis
