# Supplementary material for: Telemonitoring in adolescents with inflammatory bowel disease: a systematic review
Source: Eur J Pediatr. 2025 Aug 5;184(8):531. doi: 10.1007/s00431-025-06341-z (PMC12325510; doi:10.1007/s00431-025-06341-z)
Supplement: Supplementary file 1 — Supplementary Material 1 (DOCX 1.28 MB) [file 431_2025_6341_MOESM1_ESM.docx]

Supplementary Information

**Article title:** Telemonitoring in adolescents with inflammatory bowel disease: a systematic review

**Journal name:** European Journal of Pediatrics

**Author names:** Mike P.T. Kusters^a^*, MSc; Marleen Bouhuys^bc^*, MD PhD; Robin W.M. Vernooij^ad^, PhD; Linde F. Huis in ‘t Veld^a^, MSc; Johan E. van Limbergen^e^, MD PhD; Bada Yang^a^**, MD PhD; Patrick F. van Rheenen^b^**, MD PhD. *joint first author / **joint last author

**Affiliations:**  ^a^Cochrane Netherlands, Julius Center for Health Sciences and Primary Care, University Medical Centre Utrecht, Utrecht University, the Netherlands; ^b^Department of Pediatric Gastroenterology, Hepatology and Nutrition, University of Groningen, University Medical Centre Groningen, Beatrix Children’s Hospital, Groningen, the Netherlands; ^c^Department of Pediatrics, Frisius Medical Centre, Leeuwarden, the Netherlands; ^d^Department of Nephrology and Hypertension, University Medical Center Utrecht, Utrecht, the Netherlands; and, ^e^Department of Pediatric Gastroenterology and Nutrition, Emma Children's Hospital, Amsterdam University Medical Centers, Amsterdam, the Netherlands.

Corresponding author: p.f.van.rheenen@umcg.nl

Table of contents:

- Supplementary Table 1. Full search strategy Embase, 15 th June 2025
- Supplementary Table 2. Full search strategy MEDLINE, 15th June 2025
- Supplementary Table 3. List of excluded studies after full-text screening and reasons for exclusion
- Supplementary Table 4. Characteristics of included studies
- Supplementary Table 5. Study outcomes
- Supplementary Table 6. Results
- Supplementary Table 7. Risk of bias 2
- Supplementary Table 8. PRISMA 2020 checklist

**Supplementary Table 1.** Full search strategy Embase, 15th June 2025.

| 1 | 'inflammatory bowel disease'/exp OR 'inflammatory bowel disease' OR (('inflammatory bowel' NEAR/3 dis*):ti,ab,kw) OR ibd:ti,ab,kw OR crohn*:ti,ab,kw OR 'colitis ulcer*':ti,ab,kw OR 'ulcerative col*':ti,ab,kw OR 'idiopathic proctocol*':ti,ab,kw OR 'colitis gravis':ti,ab,kw OR 'regional enteritis':ti,ab,kw OR 'ulcerative proctocol*':ti,ab,kw OR 'mucosal colitis':ti,ab,kw OR 'ulcerous colit*':ti,ab,kw OR ((granulomatous NEAR/3 (ileit* OR enteriti*)):ti,ab,kw) OR ileocolit*:ti,ab,kw | 289.526 |
| --- | --- | --- |
| 2 | 'telehealth'/exp OR 'telemedicine'/exp OR 'telemonitoring'/exp OR 'medical informatics'/exp OR 'web-based intervention'/exp OR 'wearable sensor'/exp OR 'personal digital assistant'/exp OR 'social media'/exp OR 'e-health':ti,ab,kw OR 'ehealth':ti,ab,kw OR telehealth:ti,ab,kw OR 'tele medicine':ti,ab,kw OR 'telemedicine':ti,ab,kw OR (((tele OR distance OR remote OR patient OR tight) NEAR/3 monitor*):ti,ab,kw) OR 'mobile app*':ti,ab,kw OR ((('clinical informat*' OR 'medical informat*' OR mobile) NEAR/2 technolog*):ti,ab,kw) OR econsult*:ti,ab,kw OR 'e-consult*':ti,ab,kw OR ediagnos*:ti,ab,kw OR 'e-diagnos*':ti,ab,kw OR 'mobile-health*':ti,ab,kw OR mhealth*:ti,ab,kw OR 'm-health*':ti,ab,kw OR telehealth*:ti,ab,kw OR 'tele-health':ti,ab,kw OR telerehabilitat*:ti,ab,kw OR 'remote-consult*':ti,ab,kw OR teleconsult*:ti,ab,kw OR 'tele-consult*':ti,ab,kw OR 'video-consult*':ti,ab,kw OR videoconsult*:ti,ab,kw OR telenursing:ti,ab,kw OR (((tele OR remote OR distan*) NEAR/2 (rehabilitat* OR nurs* OR diagnos* OR medic* OR monitor* OR care OR counsel* OR consult*)):ti,ab,kw) OR telediagnos*:ti,ab,kw OR telemedic*:ti,ab,kw OR telemonitor*:ti,ab,kw OR ehealth*:ti,ab,kw OR 'e-health*':ti,ab,kw OR telecare:ti,ab,kw OR 'digital-health*':ti,ab,kw OR 'digital-intervention*':ti,ab,kw OR 'health-app*':ti,ab,kw OR telecounsel*:ti,ab,kw OR 'e-coach*':ti,ab,kw OR echoach*:ti,ab,kw OR app:ti,ab,kw | 373.477 |
| 3 | 'adolescent'/exp OR 'baby'/exp OR 'boy'/exp OR 'child'/exp OR 'minors'/exp/mj OR 'pediatric patient'/exp OR 'pediatrics'/exp OR 'schoolchild'/exp OR infan*:ti,ab OR newborn*:ti,ab OR 'new born*':ti,ab OR perinat*:ti,ab OR neonat*:ti,ab OR baby*:ti,ab OR babies:ti,ab OR toddler*:ti,ab OR minors*:ti,ab OR boy:ti,ab OR boys:ti,ab OR boyfriend:ti,ab OR boyhood:ti,ab OR girl*:ti,ab OR kid:ti,ab OR kids:ti,ab OR child*:ti,ab OR children*:ti,ab OR schoolchild*:ti,ab OR adolescen*:ti,ab OR juvenil*:ti,ab OR youth*:ti,ab OR teen*:ti,ab OR pubescen*:ti,ab OR pediatric*:ti,ab OR paediatric*:ti,ab OR peadiatric*:ti,ab OR school:ti,ab OR school*:ti,ab OR prematur*:ti,ab OR preterm*:ti,ab | 6.339.467 |
| 4 | #1 AND #2 | 2.571 |
| 5 | #4 NOT ('conference abstract'/it OR 'editorial'/it OR 'letter'/it OR 'note'/it) NOT (('animal'/exp OR 'animal experiment'/exp OR 'animal model'/exp OR 'nonhuman'/exp) NOT 'human'/exp) | 1.243 |
| 6 | efficacy AND of AND home AND telemonitoring AND versus AND conventional AND 'follow up' AND heida | 1 |
| 7 | 'self managed' AND ehealth AND monitoring AND in AND children AND adolescents AND with AND inflammatory AND bowel AND disease | 1 |
| 8 | telemedicine AND for AND management AND of AND inflammatory AND bowel AND disease AND myibdcoach AND a AND pragmatic, AND multicentre, AND randomised AND controlled AND trial | 1 |
| 9 | 'cost effectiveness' AND of AND 'telemedicine directed' AND specialized AND vs AND standard AND care AND for AND patients AND with AND inflammatory AND bowel AND diseases AND in AND a AND randomized AND trial AND jong | 1 |
| 10 | role AND of AND telemedicine AND in AND inflammatory AND bowel AND disease AND pang | 1 |
| 11 | exploring AND the AND challenges AND of AND implementing AND a AND 'web based' AND telemonitoring AND strategy AND for AND teenagers AND with AND inflammatory AND bowel AND disease | 1 |
| 12 | telemedicine AND for AND management AND of AND inflammatory AND bowel AND disease AND myibdcoach AND a AND pragmatic, AND multicentre, AND randomised AND controlled AND trial AND jong | 1 |
| 13 | . AND integration AND of AND ehealth AND into AND pediatric AND inflammatory AND bowel AND disease AND care AND is AND safe AND carlsen | 1 |
| 14 | #6 OR #7 OR #8 OR #9 OR #10 OR #11 OR #12 OR #13 | 7 |
| 15 | #5 AND #14 | 7 |
| 16 | 'meta analysis'/exp OR 'meta analysis (topic)'/exp OR metaanaly*:ti,ab OR 'meta analy*':ti,ab OR metanaly*:ti,ab OR 'systematic review'/de OR 'cochrane database of systematic reviews'/jt OR prisma:ti,ab OR prospero:ti,ab OR (((systemati* OR scoping OR umbrella OR 'structured literature') NEAR/3 (review* OR overview*)):ti,ab) OR ((systemic* NEAR/1 review*):ti,ab) OR (((systemati* OR literature OR database* OR 'data base*') NEAR/10 search*):ti,ab) OR (((structured OR comprehensive* OR systemic*) NEAR/3 search*):ti,ab) OR (((literature NEAR/3 review*):ti,ab) AND (search*:ti,ab OR database*:ti,ab OR 'data base*':ti,ab)) OR (('data extraction':ti,ab OR 'data source*':ti,ab) AND 'study selection':ti,ab) OR ('search strategy':ti,ab AND 'selection criteria':ti,ab) OR ('data source*':ti,ab AND 'data synthesis':ti,ab) OR medline:ab OR pubmed:ab OR embase:ab OR cochrane:ab OR (((critical OR rapid) NEAR/2 (review* OR overview* OR synthes*)):ti) OR ((((critical* OR rapid*) NEAR/3 (review* OR overview* OR synthes*)):ab) AND (search*:ab OR database*:ab OR 'data base*':ab)) OR metasynthes*:ti,ab OR 'meta synthes*':ti,ab | 1.153.390 |
| 17 | 'randomized controlled trial'/exp OR random*:ti,ab OR (((pragmatic OR practical) NEAR/1 'clinical trial*'):ti,ab) OR ((('non inferiority' OR noninferiority OR superiority OR equivalence) NEAR/3 trial*):ti,ab) OR rct:ti,ab,kw | 2.605.066 |
| 18 | 'major clinical study'/de OR 'clinical study'/de OR 'case control study'/de OR 'family study'/de OR 'longitudinal study'/de OR 'retrospective study'/de OR 'prospective study'/de OR 'comparative study'/de OR 'cohort analysis'/de OR ((cohort NEAR/1 (study OR studies)):ab,ti) OR (('case control' NEAR/1 (study OR studies)):ab,ti) OR (('follow up' NEAR/1 (study OR studies)):ab,ti) OR (observational NEAR/1 (study OR studies)) OR ((epidemiologic NEAR/1 (study OR studies)):ab,ti) OR (('cross sectional' NEAR/1 (study OR studies)):ab,ti) | 9.086.528 |
| 19 | 'case control study'/de OR 'comparative study'/exp OR 'control group'/de OR 'controlled study'/de OR 'controlled clinical trial'/de OR 'crossover procedure'/de OR 'double blind procedure'/de OR 'phase 2 clinical trial'/de OR 'phase 3 clinical trial'/de OR 'phase 4 clinical trial'/de OR 'pretest posttest design'/de OR 'pretest posttest control group design'/de OR 'quasi experimental study'/de OR 'single blind procedure'/de OR 'triple blind procedure'/de OR (((control OR controlled) NEAR/6 trial):ti,ab,kw) OR (((control OR controlled) NEAR/6 (study OR studies)):ti,ab,kw) OR (((control OR controlled) NEAR/1 active):ti,ab,kw) OR 'open label*':ti,ab,kw OR (((double OR two OR three OR multi OR trial) NEAR/1 (arm OR arms)):ti,ab,kw) OR ((allocat* NEAR/10 (arm OR arms)):ti,ab,kw) OR placebo*:ti,ab,kw OR 'sham-control*':ti,ab,kw OR (((single OR double OR triple OR assessor) NEAR/1 (blind* OR masked)):ti,ab,kw) OR nonrandom*:ti,ab,kw OR 'non-random*':ti,ab,kw OR 'quasi-experiment*':ti,ab,kw OR crossover:ti,ab,kw OR 'cross over':ti,ab,kw OR 'parallel group*':ti,ab,kw OR 'factorial trial':ti,ab,kw OR ((phase NEAR/5 (study OR trial)):ti,ab,kw) OR ((case* NEAR/6 (matched OR control*)):ti,ab,kw) OR ((match* NEAR/6 (pair OR pairs OR cohort* OR control* OR group* OR healthy OR age OR sex OR gender OR patient* OR subject* OR participant*)):ti,ab,kw) OR ((propensity NEAR/6 (scor* OR match*)):ti,ab,kw) OR versus:ti OR vs:ti OR compar*:ti OR ((compar* NEAR/1 study):ti,ab,kw) OR (('major clinical study'/de OR 'clinical study'/de OR 'cohort analysis'/de OR 'observational study'/de OR 'cross-sectional study'/de OR 'multicenter study'/de OR 'correlational study'/de OR 'follow up'/de OR cohort*:ti,ab,kw OR 'follow up':ti,ab,kw OR followup:ti,ab,kw OR longitudinal*:ti,ab,kw OR prospective*:ti,ab,kw OR retrospective*:ti,ab,kw OR observational*:ti,ab,kw OR 'cross sectional*':ti,ab,kw OR cross?ectional*:ti,ab,kw OR multicent*:ti,ab,kw OR 'multi-cent*':ti,ab,kw OR consecutive*:ti,ab,kw) AND (group:ti,ab,kw OR groups:ti,ab,kw OR subgroup*:ti,ab,kw OR versus:ti,ab,kw OR vs:ti,ab,kw OR compar*:ti,ab,kw OR 'odds ratio*':ab OR 'relative odds':ab OR 'risk ratio*':ab OR 'relative risk*':ab OR 'rate ratio':ab OR aor:ab OR arr:ab OR rrr:ab OR ((('or' OR 'rr') NEAR/6 ci):ab))) | 16.617.431 |
| 20 | #5 AND #16 | 145 |
| 21 | #5 AND #17 | 222 |
| 22 | #5 AND (#18 OR #19) | 757 |
| 23 | #21 NOT #20 | 193 |
| 24 | #22 NOT #21 NOT #20 | 511 |
| 25 | #20 OR #23 OR #24 | 849 |

**Supplementary Table 2.** Full search strategy MEDLINE, 15th June 2025.

| 1 | exp *Telemedicine/ or exp *Medical Informatics/ or *Internet-Based Intervention/ or exp *Wearable Electronic Devices/ or e-health.ti,ab,kf. or ehealth.ti,ab,kf. or telehealth.ti,ab,kf. or tele medicine.ti,ab,kf. or telemedicine.ti,ab,kf. or ((tele or distance or remote or patient) adj3 monitoring).ti,ab,kf. or mobile app*.ti,ab,kf. or ((clinical informat* or medical informat* or mobile or health) adj2 technolog*).ti,ab,kf. or econsult*.ti,ab,kf. or e-consult*.ti,ab,kf. or ediagnos*.ti,ab,kf. or e-diagnos*.ti,ab,kf. or mobile-health*.ti,ab,kf. or mhealth*.ti,ab,kf. or m-health*.ti,ab,kf. or telehealth*.ti,ab,kf. or tele-health.ti,ab,kf. or telerehabilitat*.ti,ab,kf. or remote-consult*.ti,ab,kf. or teleconsult*.ti,ab,kf. or tele-consult*.ti,ab,kf. or video-consult*.ti,ab,kf. or videoconsult*.ti,ab,kf. or telenursing.ti,ab,kf. or ((tele or remote or distan*) adj2 (rehabilitat* or nurs* or diagnos* or medic* or monitor* or care or counsel* or consult*)).ti,ab,kf. or telediagnos*.ti,ab,kf. or telemedic*.ti,ab,kf. or telemonitor*.ti,ab,kf. or ehealth*.ti,ab,kf. or e-health*.ti,ab,kf. or telecare.ti,ab,kf. or digital-health*.ti,ab,kf. or digital-intervention*.ti,ab,kf. or health-app*.ti,ab,kf. or telecounsel*.ti,ab,kf. or e-coach*.ti,ab,kf. or ecoach*.ti,ab,kf. | 412.968 |
| --- | --- | --- |
| 2 | exp Inflammatory Bowel Diseases/ or (inflammatory bowel adj3 dis*).ti,ab,kf. or ibd.ti,ab,kf. or crohn*.ti,ab,kf. or colitis ulcer*.ti,ab,kf. or ulcerative col*.ti,ab,kf. or idiopathic proctocol*.ti,ab,kf. or colitis gravis.ti,ab,kf. or regional enteritis.ti,ab,kf. or ulcerative proctocol*.ti,ab,kf. or mucosal colitis.ti,ab,kf. or ulcerous colit*.ti,ab,kf. or (granulomatous adj3 (ileit* or enteriti*)).ti,ab,kf. or ileocolit*.ti,ab,kf. | 157.609 |
| 3 | 1 and 2 | 1.065 |
| 4 | (child* or schoolchild* or infan* or adolescen* or pediatri* or paediatr* or neonat* or boy or boys or boyhood or girl or girls or girlhood or youth or youths or baby or babies or toddler* or childhood or teen or teens or teenager* or newborn* or postneonat* or postnat* or puberty or preschool* or suckling* or picu or nicu or juvenile?).tw. | 3.188.863 |
| 5 | 3 and 4 | 129 |
| 6 | meta-analysis/ or meta-analysis as topic/ or (metaanaly* or meta-analy* or metanaly*).ti,ab,kf. or systematic review/ or cochrane.jw. or (prisma or prospero).ti,ab,kf. or ((systemati* or scoping or umbrella or "structured literature") adj3 (review* or overview*)).ti,ab,kf. or (systemic* adj1 review*).ti,ab,kf. or ((systemati* or literature or database* or data-base*) adj10 search*).ti,ab,kf. or ((structured or comprehensive* or systemic*) adj3 search*).ti,ab,kf. or ((literature adj3 review*) and (search* or database* or data-base*)).ti,ab,kf. or (("data extraction" or "data source*") and "study selection").ti,ab,kf. or ("search strategy" and "selection criteria").ti,ab,kf. or ("data source*" and "data synthesis").ti,ab,kf. or (medline or pubmed or embase or cochrane).ab. or ((critical or rapid) adj2 (review* or overview* or synthes*)).ti. or (((critical* or rapid*) adj3 (review* or overview* or synthes*)) and (search* or database* or data-base*)).ab. or (metasynthes* or meta-synthes*).ti,ab,kf. | 840.388 |
| 7 | exp randomized controlled trial/ or randomized controlled trials as topic/ or random*.ti,ab. or rct?.ti,ab. or ((pragmatic or practical) adj "clinical trial*").ti,ab,kf. or ((non-inferiority or noninferiority or superiority or equivalence) adj3 trial*).ti,ab,kf. | 1.841.554 |
| 8 | Epidemiologic studies/ or case control studies/ or exp cohort studies/ or Controlled Before-After Studies/ or Case control.tw. or cohort.tw. or Cohort analy$.tw. or (Follow up adj (study or studies)).tw. or (observational adj (study or studies)).tw. or Longitudinal.tw. or Retrospective*.tw. or prospective*.tw. or consecutive*.tw. or Cross sectional.tw. or Cross-sectional studies/ or historically controlled study/ or interrupted time series analysis/ | 5.071.127 |
| 9 | Case-control Studies/ or clinical trial, phase ii/ or clinical trial, phase iii/ or clinical trial, phase iv/ or comparative study/ or control groups/ or controlled before-after studies/ or controlled clinical trial/ or double-blind method/ or historically controlled study/ or matched-pair analysis/ or single-blind method/ or (((control or controlled) adj6 (study or studies or trial)) or (compar* adj (study or studies)) or ((control or controlled) adj1 active) or "open label*" or ((double or two or three or multi or trial) adj (arm or arms)) or (allocat* adj10 (arm or arms)) or placebo* or "sham-control*" or ((single or double or triple or assessor) adj1 (blind* or masked)) or nonrandom* or "non-random*" or "quasi-experiment*" or "parallel group*" or "factorial trial" or "pretest posttest" or (phase adj5 (study or trial)) or (case* adj6 (matched or control*)) or (match* adj6 (pair or pairs or cohort* or control* or group* or healthy or age or sex or gender or patient* or subject* or participant*)) or (propensity adj6 (scor* or match*))).ti,ab,kf. or (confounding adj6 adjust*).ti,ab. or (versus or vs or compar*).ti. or ((exp cohort studies/ or epidemiologic studies/ or multicenter study/ or observational study/ or seroepidemiologic studies/ or (cohort* or 'follow up' or followup or longitudinal* or prospective* or retrospective* or observational* or multicent* or 'multi-cent*' or consecutive*).ti,ab,kf.) and ((group or groups or subgroup* or versus or vs or compar*).ti,ab,kf. or ('odds ratio*' or 'relative odds' or 'risk ratio*' or 'relative risk*' or aor or arr or rrr).ab. or (("OR" or "RR") adj6 CI).ab.)) | 6.010.339 |
| 10 | 5 and 6 | 16 |
| 11 | 5 and 7 | 19 |
| 12 | 5 and (7 or 8) | 53 |
| 13 | 11 not 10 | 12 |
| 14 | 12 not 11 not 10 | 31 |
| 15 | 10 or 13 or 14 | 59 |

**Supplementary Table 3.** List of excluded studies after full-text screening and reasons for exclusion.

| First author and year | Title | Reason for exclusion |
| --- | --- | --- |
| Al Khoury (2022) | Patient Perspectives and Expectations in Inflammatory Bowel Disease: A Systematic Review | Wrong study design |
| Ankersen (2019) | Individualized home-monitoring of disease activity in adult patients with inflammatory bowel disease can be recommended in clinical practice: A randomized-clinical trial | Wrong intervention |
| Ankersen (2021) | Costs of electronic health vs. standard care management of inflammatory bowel disease across three years of follow-up–a Danish register-based study | Wrong intervention |
| Arrigo (2021) | Impact of COVID-19 pandemic on the management of paediatric inflammatory bowel disease: An Italian multicentre study on behalf of the SIGENP IBD Group | Wrong study design |
| Baima (2022) | Second Brazilian consensus on the management of ulcerative colitis in adults: a consensus of the Brazilian Organization for Crohn’s Disease and Colitis (GEDIIB) | Wrong publication type |
| Bensted (2022) | Gastroenterology hospital outpatients report high rates of satisfaction with a Telehealth model of care | Wrong study design |
| Bertani (2021) | Hospitalisation for drug infusion did not increase levels of anxiety and the risk of disease relapse in patients with inflammatory bowel disease during covid-19 outbreak | Wrong intervention |
| Berkanish (2022) | Technology-Based Peer Support Interventions for Adolescents with Chronic Illness: A Systematic Review | Wrong study design |
| Bilgrami (2020) | Effect of TELEmedicine for Inflammatory Bowel Disease on Patient Activation and Self-Efficacy | Wrong outcome |
| Bonnaud (2021) | Real-life pilot study on the impact of the telemedicine platform EasyMICI–MaMICI® on quality of life and quality of care in patients with inflammatory bowel disease | Wrong population |
| Carlsen (2017) | Individualized Infliximab Treatment Guided by Patient-managed eHealth in Children and Adolescents with Inflammatory Bowel Disease | Wrong study design |
| Carlsen (2021) | Integration of eHealth Into Pediatric Inflammatory Bowel Disease Care is Safe: 3 Years of Follow-up of Daily Care | Wrong study design |
| Chee (2022) | Patient-led Remote IntraCapillary pharmacoKinetic Sampling (fingerPRICKS) for Therapeutic Drug Monitoring in patients with Inflammatory Bowel Disease | Wrong intervention |
| Chudy-Onwugaje (2018) | Age modifies the association between depressive symptoms and adherence to self-testing with Telemedicine in Patients with inflammatory bowel disease | Wrong study design |
| Chugh (2023) | A Digital Health Intervention to Improve the Clinical Care of Inflammatory Bowel Disease Patients | Wrong population |
| Cross (2006) | Acceptance of telemanagement is high in patients with inflammatory bowel disease | Wrong study design |
| Cross (2012) | Randomized, controlled trial of home telemanagement in patients with ulcerative colitis (UC HAT) | Wrong population |
| Cross (2019) | A Randomized Controlled Trial of TELEmedicine for Patients with Inflammatory Bowel Disease (TELE-IBD) | Wrong population |
| Danese (2024) | Clinical Trial: A Pragmatic Randomised Controlled Study to Assess the Effectiveness of Two Patient Management Strategies in Mild to Moderate Ulcerative Colitis—The OPTIMISE Study | Wrong population |
| Del Hoyo (2019) | Telemonitoring of Crohn's Disease and Ulcerative Colitis (TECCU): Cost-Effectiveness Analysis | Wrong study design |
| De Jong (2017) | Telemedicine for management of inflammatory bowel disease (myIBDcoach): a pragmatic, multicentre, randomised controlled trial | Wrong population |
| De Jong (2020) | Cost-effectiveness of Telemedicine-directed Specialized vs Standard Care for Patients With Inflammatory Bowel Diseases in a Randomized Trial | Wrong population |
| Del Hoyo (2018) | A Web-Based Telemanagement System for Improving Disease Activity and Quality of Life in Patients With Complex Inflammatory Bowel Disease: Pilot Randomized Controlled Trial | Wrong population |
| Del Hoyo (2023) | Are we ready for telemonitoring inflammatory bowel disease? A review of advances, enablers, and barriers | Wrong study design |
| Dijkstra (2019) | Exploring the Challenges of Implementing a Web-Based Telemonitoring Strategy for Teenagers With Inflammatory Bowel Disease: Empirical Case Study | Wrong study design |
| Di Joseph (2021) | P070 Next Generation Wearable Technology for IBD Patients: A Feasibility Study | Wrong publication type |
| El Hajra (2021) | Consequences and management of COVID-19 on the care activity of an inflammatory Bowel Disease unit | Wong intervention |
| Elkjaer (2010) | E-health empowers patients with ulcerative colitis: A randomised controlled trial of the web-guided 'constant-care' approach | Wrong population |
| Elkjaer (2012) | E-Health: Web-guided therapy and disease self-management in ulcerative colitis: Impact on disease outcome, quality of life and compliance | Wrong intervention |
| Gordon (2023) | Remote care through telehealth for people with inflammatory bowel disease | Wrong study design |
| Heida (2018) | Efficacy of Home Telemonitoring versus Conventional Follow-up: A Randomized Controlled Trial among Teenagers with Inflammatory Bowel Disease | Duplicate |
| Hommel (2023) | Digital Therapeutic Self-Management Intervention in Adolescents with Inflammatory Bowel Disease | Wrong study design |
| Huang (2014) | Distance management of inflammatory bowel disease: Systematic review and meta-analysis | Wrong study design |
| Iizawa (2023) | A Systematic Review of Self-Management Interventions for Patients with Inflammatory Bowel Disease | Wrong study design |
| Imbrizi (2023) | SECOND BRAZILIAN CONSENSUS ON THE MANAGEMENT OF CROHN'S DISEASE IN ADULTS: A CONSENSUS OF THE BRAZILIAN ORGANIZATION FOR CROHN'S DISEASE AND COLITIS (GEDIIB) | Wrong publication type |
| Jackson (2016) | EHealth technologies in inflammatory bowel disease: A systematic review | Wrong comparator |
| Jogendran (2023) | Optimizing maternal and neonatal outcomes through tight control management of inflammatory bowel disease during pregnancy: a pilot feasibility study | Wrong study design |
| Karimi (2021) | The effect of a nurse-led advice line and virtual clinic on inflammatory bowel disease service delivery: An Australian study | Wrong study design |
| Kim (2022) | Impact of Telehealth on Medication Adherence in Chronic Gastrointestinal Diseases | Wrong study design |
| Krier (2011) | Potential use of telemedicine to provide outpatient care for inflammatory bowel disease | Wrong intervention |
| Kuriakose (2023) | Management of Inflammatory Bowel Disease Using E-Health Technologies: A Systematic Review and Meta-Analysis | Wrong study design |
| Lindhagen (2022) | A more frequent disease monitoring but no increased disease activity in patients with inflammatory bowel disease during the first year of the SARS-CoV-2 pandemic. A retrospective study | Wrong intervention |
| Manzari (2025) | Effect of Self-Care Education on Lifestyle and Quality of Life in Patients With Inflammatory Bowel Diseases: A Comparison Between a Smartphone Application and Teach-Back | Wrong population |
| Marín-Jiménez (2016) | Diagnostic performance of the simple clinical colitis activity index self-administered online at home by patients with ulcerative colitis: CRONICA-UC study | Wrong study design |
| Matini (2023) | Development of the Escalation of Therapy or Intervention (ETI) calculator for patients with ulcerative colitis using ePROMS | Wrong study design |
| McCombie (2020) | A Noninferiority Randomized Clinical Trial of the Use of the Smartphone-Based Health Applications IBDsmart and IBDoc in the Care of Inflammatory Bowel Disease Patients | Wong population |
| Menze (2023) | KARLOTTA (Kids + Adolescents Research Learning on Tablet Teaching Aachen) randomized controlled pilot study for the implementation of a digital educational app with game of skill for pediatric patients with inflammatory bowel disease | Wrong intervention |
| Menze (2023) | KARLOTTA (Kids + Adolescents Research Learning on Tablet Teaching Aachen) randomized controlled pilot study for the implementation of a digital educational app with game of skill for pediatric patients with inflammatory bowel disease | Duplicate |
| Michel (2023) | Impact of Telemedicine on Delivery of Pediatric Inflammatory Bowel Disease Care | Wrong study design |
| Miloh (2017) | Text messaging effect on adherence in children with inflammatory bowel disease | Wrong intervention |
| Nguyen (2022) | Digital Health Technologies for Remote Monitoring and Management of Inflammatory Bowel Disease: A Systematic Review | Wrong study design |
| Nielsen (2022) | Readiness, acceptance and use of digital patient reported outcome in an outpatient clinic | Wrong intervention |
| Noser (2023) | Use of Behavior Change Techniques and Quality of Commercially Available Inflammatory Bowel Disease Apps | Wrong study design |
| Östlund (2021) | Self-monitoring with home based fecal calprotectin is associated with increased medical treatment. A randomized controlled trial on patients with inflammatory bowel disease | Wrong population |
| Pang (2022) | Role of Telemedicine in Inflammatory Bowel Disease: Systematic Review and Meta-analysis of Randomized Controlled Trials | Wrong study design |
| Quinn (2019) | The Telemedicine for Patients With Inflammatory Bowel Disease (TELE-IBD) Clinical Trial: Qualitative Assessment of Participants' Perceptions | Wrong study design |
| Ramelet (2017) | Impact of a nurse led telephone intervention on satisfaction and health outcomes of children with inflammatory rheumatic diseases and their families: A crossover randomized clinical trial | Wrong population |
| Ramos (2023) | Efficacy, Efficiency, and Acceptability of Telemedicine for Inflammatory Bowel Disease Patients' Follow-Up Care during the COVID-19 Pandemic | Wrong study design |
| Schliep (2020) | TELEmedicine for patients with inflammatory bowel disease (tele-IBD) does not improve depressive symptoms or general quality of life compared with standard care at tertiary referral centers | Secondary analysis (outcome: depression) of Cross (2019) |
| Shah (2021) | Telehealth model of care for outpatient inflammatory bowel disease care in the setting of the COVID-19 pandemic | Wrong study design |
| Srinivasan (2020) | A virtual clinic increases anti-TNF dose intensification success via a treat-to-target approach compared with standard outpatient care in Crohn’s disease | Wrong study design |
| Stone (2023) | Risk factors for incomplete telehealth appointments among patients with inflammatory bowel disease | Wrong study design |
| Yao (2022) | Telemonitoring for patients with inflammatory bowel disease amid the COVID-19 pandemic—A cost-effectiveness analysis | Wrong intervention |
| Yilmaz (2021) | Smartphone-based videoconference visits are easy to implement, effective, and feasible in Crohn's Disease Patients: A prospective cohort study | Wrong study design |
| Yilmaz (2021) | Smartphone-based videoconference visits are easy to implement, effective, and feasible in Crohn's Disease Patients: A prospective cohort study | Duplicate |
| Zand (2020) | Patient Experiences and Outcomes of a Telehealth Clinical Care Pathway for Postoperative Inflammatory Bowel Disease Patients | Wrong study design |
| Zhang (2021) | Impact of COVID-19 outbreak on the care of patients with inflammatory bowel disease: A comparison before and after the outbreak in South China | Wrong study design |
| Zhen (2024) | Acceptability, feasibility, and impact of the MyGut digital health platform in the monitoring and management of inflammatory bowel disease | Wrong study design |

**Supplementary Table 4.** Characteristics of included studies.

| Study | Study characteristics | Patient characteristics | Telemonitoring type and intervention interval (I) | Comparison / control and time interval (C) | Follow-up (months) | Outcome measures |
| --- | --- | --- | --- | --- | --- | --- |
| Akobeng et al., 2015^17^ | **Design:** Parallel RCT    Participants randomized (n):  86 (44 intervention, 42 control)    **Conflicts of interest/Funding:** Funded by Research for Patient Benefit Programme,  UK National Institute for Health Research | Median age at enrolment in years [IQR]:  I: 13·9[12·1-15·9] C:13·8[11·2-15·3]  **Female (%):** 37  **Disease type (%):**  UC: 17; CD: 83  Active disease at enrolment (%): 0 | **Telemonitoring type:** Phone-based (planned telephone consultations)  Intervention interval: NR | Regular face-to-face consultation: Planned outpatient visits  Time interval: NR | 24 | **Disease activity:** disease relapses (≥1 event(s) PUCAI or aPCDAI score >15)    **QoL:** pediatric IBD-IMPACT QoL    **Costs:** costs to the UK National Health Service (NHS)    **Patient satisfaction:** child-modified consultation satisfaction (CSQ-child)    **Patient adherence:** consultation attendance  **Face-to-face outpatient contacts:** number of contacts in the control group |
| Carlsen et al., 2017^18^ | **Design:** Parallel RCT    **Participants randomized (n):** 53 (27 intervention, 26 control)  **Conflicts of interest/funding:** work was supported by the European Crohn’s and  Colitis Organization, Queen Louise’s Hospital Foundation, Tryg  Foundation, CALPRO A/S, Tillotts Pharma, Capital Region  Denmark, Alice and Frimodts Foundation, Ulcerative colitis and  Crohn’s Danish Patient Society, and Merck Sharp and Dome. | Mean age at enrolment in years (SD): I: 15·1 (1·82) C: 14·7 (2·11)  Female (%): 58  Disease type (%): UC: 60; CD: 40  Active disease at enrolment (%): 57 | Telemonitoring type:  Web-based (automated text message alerts for data entry in a web application)  **Intervention interval:** Monthly use of web application, one face-to-face consultation per year. Blood and fecal calprotectin test every 3 months. | Regular face-to-face consultation: Planned outpatient visits    Time interval:  Every third month. Blood and fecal calprotectin test every 3 months. | 24 | **Disease activity:** step up in treatment intensity    **QoL:** pediatric IBD-IMPACT III    **Patient adherence:** medication adherence (MARS and VAS)  **Face-to-face outpatient contacts:** outpatient visits (planned/on-demand/total)    **Unplanned ER/surgery/hospitalizations:** acute (unplanned emergency room) outpatient visits and hospitalizations |
| Heida et al., 2018^16^ | **Design:** Parallel RCT    Participants randomized (n):  170 (84 intervention, 86 control)  **Conflicts of interest/funding:** work was supported by ZonMw Health Care Efficiency Research, Innovation Fund Dutch Insurance Companies NutsOhra Fund, and Ferring Pharmaceuticals BV. Author(s) supported by  Netherlands Organization for Scientific Research, BÜHLMANN Laboratories and CisBio Bioassays. | Median age at enrolment in years [IQR]: I: 15 [12-16]  C: 15 [13-17]   Female (%): 54·7   Disease type (%):  UC: 52·4; CD: 47·6   Active disease at enrolment (%): 0 | Telemonitoring type:  Web-based (automated email alerts for filling in symptom scores and submitting a stool sample)  **Intervention interval:** Based on risk stratification. Low risk participants: retest in 3 months.  Intermediate-risk participants: subjected to a shorter test interval before progressing to a decision.  High-risk participants: advised to contact their specialist.  In addition, all participants had regular face-to-face consultation every 6 months. | **Regular face-to-face consultation:** Planned outpatient visits and emergency visits.    Time interval:  Regular checks regardless of disease activity and interval varied according to the physician’s discretion. | 12 | **Disease activity:** disease flares (cumulative incidence)    **QoL:** IBD-specific IMPACT-III    **Costs:** all direct and indirect medical and non-medical costs    **Patient satisfaction:** participants’ opinion about home telemonitoring (Likert-scale based questionnaire)    **Patient adherence:** adherence to study protocol (intervention: response to 80% of automated alerts; control: 2 requested stool samples for calprotectin measurement)  **Face-to-face outpatient contacts:** outpatient visits, face-to-face care provider encounters  Unplanned ER/surgery/hospitalizations: emergency consultations |

aPCDAI: abbreviated Pediatric Crohn Disease Activity Index; C: control group; CD: Crohn’s disease; CSQ-child: child-modified consultation satisfaction questionnaire; IBD-IMPACT: IBD quality of life questionnaire; I: intervention group; IQR: interquartile range; n: number of patients; NHS: National Health Service; MARS: Medication Adherence Report Scale; NR: not reported; PCDAI: Pediatric Crohn Disease Activity Index; PUCAI: Pediatric Ulcerative Colitis Activity Index; QoL: quality of life; RCT: randomized controlled trial; SD: standard deviation; UC: ulcerative colitis; VAS: Visual Analog Scale

**Supplementary Table 5.** Study outcomes.

| Study name | Disease activity (flares, treatment intensity, etc.) | QoL | Costs (any kind) | Patient satisfaction and adherence | Face-to-face outpatient contacts | Unplanned ER/surgery/hospitalizations |
| --- | --- | --- | --- | --- | --- | --- |
| Akobeng et al., 2015^17^ | Count ;  Disease relapse (≥1 event(s) PUCAI or aPCDAI score >15) | Continuous ;  Paediatric IBD-IMPACT | Continuous; Costs to NHS per consultation | Continuous ;  CSQ-child (Patient satisfaction)  Binary: Proportion of consultations attended (Patient adherence) | Count ; median (IQR) and proportion | NR |
| Carlsen et al., 2017^18^ | Time to event;  Treatment intensity step-up | Continuous ;  Paediatric IBD-IMPACT III | NR | Continuous ;  MARS and adherence VAS (Self-rated medication adherence) | Count ;  Planned, on-demand, and total outpatient visits | Count ;  Acute outpatient visits/hospitalizations |
| Heida et al., 2018^16^ | Binary;  Cumulative incidence of disease flares (disease activity requiring treatment intensification) | Continuous **;**  IBD-specific IMPACT-III (change from baseline) | Direct and indirect medical and non-medical costs | Binary: Proportion considering telemonitoring as timesaving (Patient satisfaction).  Proportion wishing to continue with telemonitoring (Patient satisfaction).  Proportion adhering to study protocol, defined as response to ≥80% of email alerts (intervention group) or ≥2 fecal calprotectin results (control group) (Patient adherence). | Count; mean per patient | Count |

aPCDAI: abbreviated Pediatric Crohn Disease Activity Index; CSQ-child: child-modified consultation satisfaction questionnaire; IBD-IMPACT: IBD quality of life questionnaire; IQR: interquartile range; MARS: Medication Adherence Report Scale NR: not reported. Outcome has not been incorporated into the study; PCDAI: Pediatric Crohn Disease Activity Index; PUCAI: Pediatric Ulcerative Colitis Activity Index; QoL: Quality of life; VAS: Visual Analog Scale

**Supplementary Table 6.** Results.

| Study name | Disease activity (flares, relapses, treatment intensity change ) | QoL | Costs (any kind) | Patient satisfaction and adherence | Face-to-face outpatient contacts | Unplanned ER/surgery/hospitalizations |
| --- | --- | --- | --- | --- | --- | --- |
| Akobeng et al., 2015^17^ | Disease relapses. Events (n ), %:  I: 1 (n=44), 2%  C: 4 (n=42), 10%  p=0·20    RR for disease relapse: 0.24 (0.03 to 2·05), p=0·20 | Pediatric IBD-IMPACT score.  Median[IQR], n:  I: 113[105-125], n=31  C: 106[95-116], n=36    †Mean (SD), n:  I: 114·3 (15·5 ), n=31  C: 105·7 (16·2 ), n=36    MD: 8·7 (1·1 to 16·3 ), p=0.03    Adjusted MD (95% CI): 5·7 (-2·9, 14·3)*  p=0·19    * Baseline outcome, disease type included as covariates. | Mean costs in UK pounds per consultation (n):  I: 35·4 (n=44)  C: 51·1 (n=42)    MD: -15·7; 95% CI: -11·8 to -19·6, p<0·001 | CSQ-child (Patient satisfaction).  Median[IQR], n:  I : 48[45; 51], n=36  C : 46[44; 51], n=40    †Mean (SD), n:  I : 48 (4·63), n=36  C : 47 (5·38), n=40      MD : 1·00 (-1·25 to 3·25), p=0.38      Adjusted MD (95%, CI): 0.59 (-2·05 to 3·24)  p = .65    Proportion of consultations attended (Patient adherence).  I: 67% (n=43)  C: 71% (n=42)  RR for attendance: 1.06 (0.78 to 1.43), p=0.71  * Baseline outcome, disease type included as covariates. | Consultations per patient. Median [IQR].  I: NA  C: 3 [2 to 4] | NR |
| Carlsen et al., 2017^18^ | Step-up treatment intensity. Median time to event (total patients);  I: NR (n=15)  C: NR (n=18)  Log rank test: p=0·53 | Pediatric IBD-IMPACT III score.  No difference between groups; adjusted for baseline age and sex. | NR | Medication adherence (MARS and VAS).  No difference between groups; adjusted for age and time from diagnosis. | Planned outpatient visits. Count ; Median [IQR], n:  I: 38; 2 [1 to 2], n=15  C: 146; 7 [3 to 7], n=18  p<0·0001    On-demand outpatient visits. Count ; Median [IQR], n:  I: 47; 1 [0 to 3], n=15  C: 39; 1 [0 to 2], n=18  p=0.68  Total outpatient visits. Count ; m edian [IQR], n:  I: 85; 2 [2 to 3], n=15  C: 185; 8 [4 to 9], n=18  p<0·0001 | Acute outpatient visits and hospitalizations. Count; median [IQR], n:  I: 3; 0 [0 to 0 ], n=15  C: 10; 0 [0 to 1 ], n=18  p=0·13 |
| Heida et al., 2018^16^ | D isease flares .  Events (n ), %:  I: 28 (n=84), 33%  C: 29 (n=86), 34%    RR for disease flare: 0.99 (0.65 to 1·51), p=0.96  Log rank test for difference in time to disease flare: 0.93 | IBD-specific IMPACT III score.  Mean change score from baseline, n:  I: +1·32, n=84  C: -0.32, n=86  p=0·27 | Annual cost-saving per participant telemonitoring versus conventional follow-up (euro): 89 to 360 in those compliant to protocol. | Telemonitoring satisfaction (59 of 84 (70%) telemonitoring group patients ).   - 96% of respondents agreed that home telemonitoring is timesaving. - 71% wished to continue with home telemonitoring .     Adherence to study protocol, Events (n ), %.  I: 48 (n=84), 57%  C: 72 (n=86), 84%  RR for non-adherence: 2·63 (1·54 to 4·51), p=0·0004 | Outpatient visits, count :  I: 300  C: 328  [Mean per patient. I: 3·6  C: 4·3  p<0·001 . | Emergency consultations, count:  I: 4  C: 5  No measures of precision reported. |

All effect estimates are calculated as intervention minus or divided by control. Where applicable, medians (IQR and ranges) were converted to means (SD) using the formulas by Wan et al.[15] †Median[IQR] scores converted to mean (SD) scores using standardized formulas by Wan et al.[15]

Abbreviations. C: control group; CI: confidence interval; CSQ-child: child-modified consultation satisfaction questionnaire; I: intervention group; IBD-Impact: IBD quality of life questionnaire; IQR: interquartile range; MARS: Medication Adherence Report Scale; MD: mean difference; n: number of patients; NR: not reported; QoL: quality of life; RR: risk ratio; SD: standard deviation; SMD: standardized mean difference; VAS: Visual Analog Scale

**Supplementary Table 7.** Risk of bias 2.^10^

|  | Randomization process | Deviations from intended interventions | Missing outcome data | Measurement of the outcome* | Selection of the reported result | Overall |
| --- | --- | --- | --- | --- | --- | --- |
| Akobeng et al., 2015^17^ | + | + | + | ? | + | ? |
| Carlsen et al., 2017^18^ | + | + | - | ? | + | - |
| Heida et al., 2018^16^ | + | + | + | ? | + | ? |

+ Low risk of bias; ? Some concerns of bias; - High risk of bias

*Low risk of bias for costs, face-to-face outpatient contacts, and the unplanned ER (emergency room) visits, surgery and hospitalizations outcome domain

**Supplementary Table 8.** PRISMA 2020 checklist

| Section and Topic | Item # | Checklist item | Location where item is reported |
| --- | --- | --- | --- |
| TITLE | | |  |
| Title | 1 | Identify the report as a systematic review. | Title |
| ABSTRACT | | |  |
| Abstract | 2 | See the PRISMA 2020 for Abstracts checklist. | Abstract |
| INTRODUCTION | | |  |
| Rationale | 3 | Describe the rationale for the review in the context of existing knowledge. | Introduction, paragraph 1-3 |
| Objectives | 4 | Provide an explicit statement of the objective(s) or question(s) the review addresses. | Introduction, paragraph 4 |
| METHODS | | |  |
| Eligibility criteria | 5 | Specify the inclusion and exclusion criteria for the review and how studies were grouped for the syntheses. | Search strategy and selection criteria |
| Information sources | 6 | Specify all databases, registers, websites, organizations, reference lists and other sources searched or consulted to identify studies. Specify the date when each source was last searched or consulted. | Search strategy and selection criteria |
| Search strategy | 7 | Present the full search strategies for all databases, registers and websites, including any filters and limits used. | Search strategy and selection criteria, Supplementary Table 1 and 2 |
| Selection process | 8 | Specify the methods used to decide whether a study met the inclusion criteria of the review, including how many reviewers screened each record and each report retrieved, whether they worked independently, and if applicable, details of automation tools used in the process. | Search strategy and selection criteria |
| Data collection process | 9 | Specify the methods used to collect data from reports, including how many reviewers collected data from each report, whether they worked independently, any processes for obtaining or confirming data from study investigators, and if applicable, details of automation tools used in the process. | Data extraction and assessment of study quality |
| Data items | 10a | List and define all outcomes for which data were sought. Specify whether all results that were compatible with each outcome domain in each study were sought (e.g. for all measures, time points, analyses), and if not, the methods used to decide which results to collect. | Introduction, Data extraction and assessment of study quality |
|  | 10b | List and define all other variables for which data were sought (e.g. participant and intervention characteristics, funding sources). Describe any assumptions made about any missing or unclear information. | Data extraction and assessment of study quality |
| Study risk of bias assessment | 11 | Specify the methods used to assess risk of bias in the included studies, including details of the tool(s) used, how many reviewers assessed each study and whether they worked independently, and if applicable, details of automation tools used in the process. | Data extraction and assessment of study quality |
| Effect measures | 12 | Specify for each outcome the effect measure(s) (e.g. risk ratio, mean difference) used in the synthesis or presentation of results. | Data analysis |
| Synthesis methods | 13a | Describe the processes used to decide which studies were eligible for each synthesis (e.g. tabulating the study intervention characteristics and comparing against the planned groups for each synthesis (item #5)). | Data analysis |
|  | 13b | Describe any methods required to prepare the data for presentation or synthesis, such as handling of missing summary statistics, or data conversions. | Data analysis |
|  | 13c | Describe any methods used to tabulate or visually display results of individual studies and syntheses. | Data analysis |
|  | 13d | Describe any methods used to synthesize results and provide a rationale for the choice(s). If meta-analysis was performed, describe the model(s), method(s) to identify the presence and extent of statistical heterogeneity, and software package(s) used. | Data analysis |
|  | 13e | Describe any methods used to explore possible causes of heterogeneity among study results (e.g. subgroup analysis, meta-regression). | NA |
|  | 13f | Describe any sensitivity analyses conducted to assess robustness of the synthesized results. | NA |
| Reporting bias assessment | 14 | Describe any methods used to assess risk of bias due to missing results in a synthesis (arising from reporting biases). | NA |
| Certainty assessment | 15 | Describe any methods used to assess certainty (or confidence) in the body of evidence for an outcome. | Data extraction and assessment of study quality |
| RESULTS | | |  |
| Study selection | 16a | Describe the results of the search and selection process, from the number of records identified in the search to the number of studies included in the review, ideally using a flow diagram. | Study selection, Fig. 1 |
|  | 16b | Cite studies that might appear to meet the inclusion criteria, but which were excluded, and explain why they were excluded. | Supplementary Table 3 |
| Study characteristics | 17 | Cite each included study and present its characteristics. | Description of studies |
| Risk of bias in studies | 18 | Present assessments of risk of bias for each included study. | Description of studies, Supplementary Table 7 |
| Results of individual studies | 19 | For all outcomes, present, for each study: (a) summary statistics for each group (where appropriate) and (b) an effect estimate and its precision (e.g. confidence/credible interval), ideally using structured tables or plots. | Results (subheadings for each outcome), Table 1 , Supplementary Tables 5 and 6 |
| Results of syntheses | 20a | For each synthesis, briefly summarize the characteristics and risk of bias among contributing studies. | Results (subheadings for each outcome) |
|  | 20b | Present results of all statistical syntheses conducted. If meta-analysis was done, present for each the summary estimate and its precision (e.g. confidence/credible interval) and measures of statistical heterogeneity. If comparing groups, describe the direction of the effect. | Results (subheadings for each outcome), Table 1 |
|  | 20c | Present results of all investigations of possible causes of heterogeneity among study results. | NA |
|  | 20d | Present results of all sensitivity analyses conducted to assess the robustness of the synthesized results. | NA |
| Reporting biases | 21 | Present assessments of risk of bias due to missing results (arising from reporting biases) for each synthesis assessed. | NA |
| Certainty of evidence | 22 | Present assessments of certainty (or confidence) in the body of evidence for each outcome assessed. | Table 2 |
| DISCUSSION | | |  |
| Discussion | 23a | Provide a general interpretation of the results in the context of other evidence. | Key findings |
|  | 23b | Discuss any limitations of the evidence included in the review. | Strengths and limitations |
|  | 23c | Discuss any limitations of the review processes used. | Strengths and limitations |
|  | 23d | Discuss implications of the results for practice, policy, and future research. | Implications for clinical practice |
| OTHER INFORMATION | | |  |
| Registration and protocol | 24a | Provide registration information for the review, including register name and registration number, or state that the review was not registered. | Methods – search strategy and selection criteria |
|  | 24b | Indicate where the review protocol can be accessed, or state that a protocol was not prepared. | Methods – search strategy and selection criteria |
|  | 24c | Describe and explain any amendments to information provided at registration or in the protocol. | Methods – Data analysis |
| Support | 25 | Describe sources of financial or non-financial support for the review, and the role of the funders or sponsors in the review. | Funding source |
| Competing interests | 26 | Declare any competing interests of review authors. | Conflict of interest |
| Availability of data, code and other materials | 27 | Report which of the following are publicly available and where they can be found: template data collection forms; data extracted from included studies; data used for all analyses; analytic code; any other materials used in the review. | Data availability statement: |
